# Poster Session I - A112 CYPROHEPTADINE USE IN CHILDREN 24 MONTHS AND YOUNGER: EFFICACY, SAFETY AND ROLE IN TUBE FEEDING WEANING

**DOI:** 10.1093/jcag/gwaf042.112

**Published:** 2026-02-13

**Authors:** B Leduc, Z Martineau-Karakach, P Joly, V Kirouak, S St-Gelais, D Nadeau-Poulin, S Cayer, M Turcotte, G Costaguta, J Castilloux, A Wieckowska

**Affiliations:** Pediatrics, Centre hospitalier universitaire de l’Université Laval, Québec, QC, Canada; Universite Laval, Québec City, QC, Canada; Pediatrics, Centre hospitalier universitaire de l’Université Laval, Québec, QC, Canada; Pediatrics, Centre hospitalier universitaire de l’Université Laval, Québec, QC, Canada; Pediatrics, Centre hospitalier universitaire de l’Université Laval, Québec, QC, Canada; Pediatrics, Centre hospitalier universitaire de l’Université Laval, Québec, QC, Canada; Pediatrics, Centre hospitalier universitaire de l’Université Laval, Québec, QC, Canada; Pediatrics, Centre hospitalier universitaire de l’Université Laval, Québec, QC, Canada; Pediatrics, Centre hospitalier universitaire de l’Université Laval, Québec, QC, Canada; Pediatrics, Centre hospitalier universitaire de l’Université Laval, Québec, QC, Canada; Pediatrics, Centre hospitalier universitaire de l’Université Laval, Québec, QC, Canada

## Abstract

**Background:**

Cyproheptadine (CY), a first-generation H1-antihistamine with antiserotonergic effects, initially developed to treat allergic conditions, has been used for its appetite stimulation. Although CY has been studied in older children, there is limited data on its use in those aged 24 months or younger. At Centre Hospitalier de l’Université Laval, we have treated children under 24 months with CY for appetite stimulation, reflux, vomiting, and tube-feeding weaning.

**Aims:**

To evaluate the safety and efficacy of CY in children aged under 24 months, and its effects on weight gain, appetite stimulation, reflux, vomiting, and tube-feeding weaning.

**Methods:**

We conducted a retrospective study (January 2015–June 2025) including patients aged 0–24 months who received CY for feeding difficulties. All patients were followed at the eating disorder clinic by the same multidisciplinary team. Patients were divided into two groups based on age at CY initiation: ≤8 months or > 8 months, using chronological age for term infants and corrected age for preterm infants. Data collected included demographics, medical history, clinical response, and adverse events.

**Results:**

Sixty-two patients (55% female) were included, 33 infants were ≤8 months old, with a mean gestational age (GA) of 34.8 ± 5 weeks and a mean birth weight (BW) of 2.25 ± 1 kg. Meanwhile, 29 young children were >8 months old, with a mean GA of 35.1 ± 5 weeks and a mean BW of 2.2 ± 1,2 kg. Mean follow-up was 11.2 months (s^2^ 47.7), slightly longer for the first group compared to the second (14.7 vs. 9.85 months, p 0.07). CY was used for appetite stimulation (94%) and for reflux or vomiting (24%), with a mean starting dose of 0.15 mg/kg/day. The median weight increased from 7.1 kg (STD 1.5) to 9.1 kg (STD 1.5), p < 0.001, with weight changes at each time point. Similar trends were observed when evaluating those ≤8 months, the median weight increased from 6.6 kg (STD 0.95) to 9.3 kg (STD 1.3), p < 0.001, and those >8 months, an increase from 7.4 kg (STD 1.7) to 9.7 kg (STD 1.8), p < 0.001. Appetite improved in 84% of patients, while reflux or vomiting improved in 67%. Furthermore, 90% of those with tube feeding achieved complete weaning, after a median of 13 ± 1 months. Mild adverse events were drowsiness/fatigue (n = 6), excitability (n = 1), irritability (n = 3), and insomnia (n = 3). No serious side effects were noted. No QT prolongation was observed. No patient discontinued the treatment.

**Conclusions:**

To our knowledge, this is the largest cohort of CY use in children under 24 months. CY was well tolerated and effective, including in premature infants as young as 6 months. It improved feeding tolerance, appetite, weight gain, and tube weaning. CY seems useful in this age group.

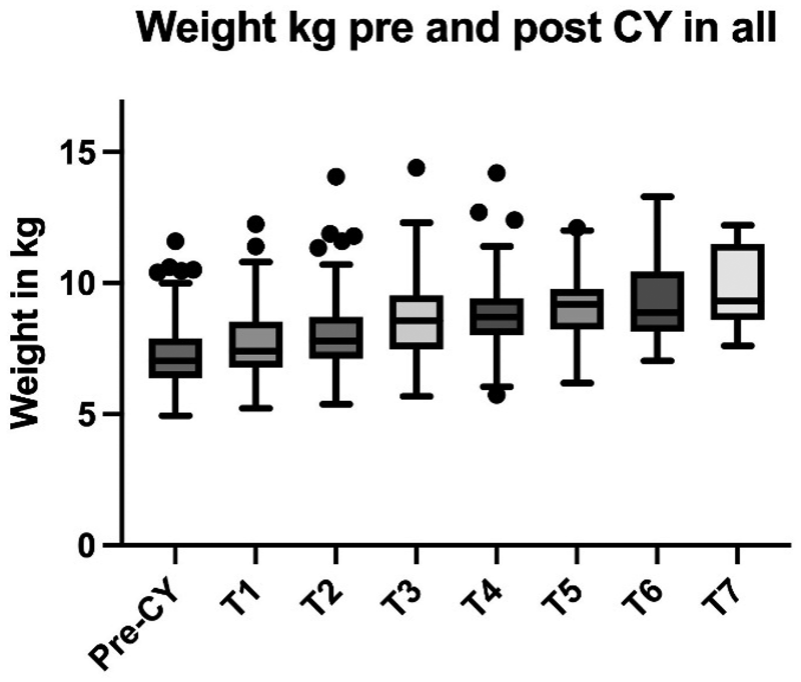

**Funding Agencies:**

None

